# Transcriptomic Analysis and Specific Expression of Transcription Factor Genes in the Root and Sporophyll of *Dryopteris fragrans* (L.) Schott

**DOI:** 10.3390/ijms21197296

**Published:** 2020-10-02

**Authors:** Lingling Chen, Dongrui Zhang, Chunhua Song, Hemeng Wang, Xun Tang, Ying Chang

**Affiliations:** College of Life Sciences, Northeast Agricultural University, Harbin 150030, Heilongjiang Province, China; chenlingling1926@gmail.com (L.C.); GeraldTwinersom@gmail.com (D.Z.); chunhuasong66@gmail.com (C.S.); Gzamopema@gmail.com (H.W.); tangxun1119@gmail.com (X.T.)

**Keywords:** transcriptome, *Dryopteris fragrans*, transcription factors, gene expression

## Abstract

Background: *Dryopteris fragrans*, which is densely covered with glandular trichomes, is considered to be one of the ferns with the most medicinal potential. The transcriptomes from selected tissues of *D. fragrans* were collected and analyzed for functional and comparative genomic studies. The aim of this study was to determine the transcriptomic characteristics of wild *D. fragrans* sporangium in tissues from the SR (root), SL (sporophyll), and TRL (sporophyll with glandular trichomes removed). Results: Cluster analysis identified genes that were highly expressed in an organ-specific manner according to read mapping, feature counting, and normalization. The functional map identified gene clusters that can uniquely describe the function of each tissue. We identified a group of three tissue-specific transcription factors targeting the SL, SR, and TRL. In addition, highly expressed transcription factors (TFs) were found in each tissue-specific gene cluster, where ERF and bHLH transcription factors were the two types showing the most distinct expression patterns between the three different tissues. The specific expression of transcription factor genes varied between the different types of tissues. The numbers of transcription factors specifically expressed in the roots and sporophylls were 60 and 30, respectively, while only seven were found for the sporophylls with glandular trichomes removed. The expression of genes known to be associated with the development of glandular trichomes in flowering plants, including MIXTA, ATML1, and MYB106, were also validated and are discussed. In particular, a unigene encoding MIXTA was identified and exhibited the highest expression level in SL in *D. fragrans.* Conclusions: This study is the first report of global transcriptomic analysis in different tissues of *D. fragrans*, and the first to discuss these findings in the context of the development of homologous glandular trichomes. These results set the stage for further research on the development, stress resistance, and secondary metabolism of *D. fragrans* glandular trichomes.

## 1. Introduction

The regulation of gene expression is one of the most significant and complicated of the various biological processes, especially in eukaryotic species, due to their large genomes and complexity in tissue organization [1,2]. Some aspects of fern morphological development have been reported, but little is known regarding the fern transcriptome. It is well-known that transcription factors (TFs) regulate gene expression by binding to specific nucleotide sequences of their target genes, and ultimately control a range of biological pathways.

*Dryopteris fragrans* is a member of the Dryopteridaceae family that is mainly located in the temperate regions of North America, Europe, and Asia [3,4]. *D. fragrans* is a valuable medicinal plant resource with extensive biological activities, including anticancer, antioxidative, insect-repellent, antimicrobial, and anti-inflammatory activities. Phytochemical investigations of the components of this plant have led to the identification of terpenoids, phloroglucinols, glucosides, and other phenolic derivatives, such as coumarin. It is characterized by multicellular, glandular trichomes with spherical head cells. Although the entire plant is densely covered with glandular trichomes, there have so far been no reports on TF genes involved in regulating their formation in *D. fragrans*.

Trichomes can be either non-glandular or glandular. Non-glandular trichomes are unable to synthesize and store secondary metabolites, while the glandular trichomes can synthesize, secrete, or store a variety of secondary metabolites, such as arteannuin, the main component in the treatment of malaria [5]. A trichome is a hairy structure that develops from the epidermal cells of plants, and can protect them against biotic and abiotic stresses, presenting both economic value and medicinal effects. The presence of trichomes can increase the thickness of the plant epidermis and reduce the loss of heat and water [5]. In many species, trichomes can hinder the attachment of insects and pathogens by secreting chemical components or acting as a physical barrier [6]. Many genes directly involved in glandular trichome development have been discovered and reviewed [7,8]. Moreover, several transcription factors have been identified as regulators in the development of the glandular trichomes, such as MYB, bHLH, AP2/ERF, and homeodomain leucine zipper class (HD-Zip) IV members [9]. However, the precise details of the regulatory network remain far from clear [10]. 

Previous studies have shown that the trichome initiation MYB–bHLH–WD40 (MBW) complex (GL3/EGL3–GL1–TTG1) plays a key role in determining the cellular fate of trichome cells. This complex triggers expression of the GLABRA2 (GL2) gene, which encodes a TF that induces the transition and differentiation of trichome cells [11,12,13]; it also functions in the development of cyanobacterial heterocysts. Besides the MBW complex, a range of genes that promote initiator–complex gene expression has been found in leaves and flowers, such as GLABROUS INFLORESCENCE STEMS 2 (GIS2) [14], zinc finger protein (ZFP) 5 [15], ZFP6, and ZFP8 [16]. Plant trichomes and cuticles both originate from the epidermis, and previous studies have shown that mutations in numerous genes, such as *Formate Dehydrogenase (FDH*), *LACERATA*, *AtSHN1*, *AmMIXTA*, *SlMIXTA-like*, and *SlCD2* exhibit abnormal cuticles and reduced trichome density, in addition to mutations in *GL1* and *GL3*, key transcription factors found in the MBW complex. There are also numerous genes involved in the regulation of trichome development, the expression of which changes the profile of metabolites in the glandular trichome; the overexpression of *AaMIXTAl*, *AaHDl*, or *AaHD8* significantly increased the secretion of artemisinin in the epidermis [16].

There are around 12,000 species of fern, which are considered to be a natural family of higher plants because of their bona fide vascular system, despite propagating and spreading via spores. Ferns adapted to a land environment during the process of evolution, which has uniquely determined their special trichome structure. There are few ferns with glandular trichomes, with which *D. fragrans* is covered. *D. fragrans* grows on talcum slopes, gravel slopes, and magmatic fissures around volcanoes at an altitude of 700–2400 m, and can survive at −20 °C. The study of glandular trichome development in *D. fragrans* is thus of great interest. We sought to determine the genes involved in trichome development in *D. fragrans* in our study by examining the regulation of trichome development genes in model higher plants as a reference. We then focused on known genes that had been characterized in model plants, such as *Arabidopsis thaliana*, in order to determine whether the trichome developmental pathway involved homologues/orthologues in ferns.

There is limited research on *D. fragrans* and the deciphering of tissue-specific genes. Transcription factors are of particular interest among the diverse types of genetic regulatory proteins, because they represent relatively direct regulatory interactions between proteins and chromosomes, which may lead to direct alterations in transcriptional activity [17,18,19]. To investigate the expression of transcription factor genes related to glandular trichome development in different tissues of *D. fragrans*, we collected three different tissue types of *D. fragrans* grown at 25 °C with a 16/8 h photoperiod over two years. We focused on the genes and transcription factors that were specific to the different tissues. Illumina sequencing is the most effective high-throughput platform for next-generation RNA-seq transcriptome analysis in non-model tissues lacking available genomic data [20]. The objective of this study was to identify the genes that showed differential expression (Supplementary File 1) in Root, Sporophyll, Sporophyll removed from glandular trichom and identified the transcription factors specifically associated with trichome development. The results provide a solid basis for future research on the development of fern trichomes.

## 2. Results

### 2.1. Sequence Analysis and Assembly

We analyzed the RNA-seq libraries for various tissues to obtain a global picture of the diversity across the tested tissues and their biological replicates (Table 1). After quality control, we obtained 54 gigabases of clean data, and the percentage Q30 (Quality Score 30) in the different samples was not less than 99.9% (Figures S1 and S2, Table S1). The correlation between the three samples was determined according to the quantitative FPKM results. In order to explore the relative variation between a repeated sample and three tissues (Figure S3), we employed hierarchical clustering to normalize all nine samples using Spearman pairwise correlation. The clustering results show similar gene expression patterns between SL and TRL. In conclusion, these assessments showed that variability in gene expression among the replicates for the same tissues was much lower than that among the three different tissues, indicative of the high quality of the dataset.

### 2.2. Global Analysis Showing Differences between the Three Tissues

**Figure 1:**
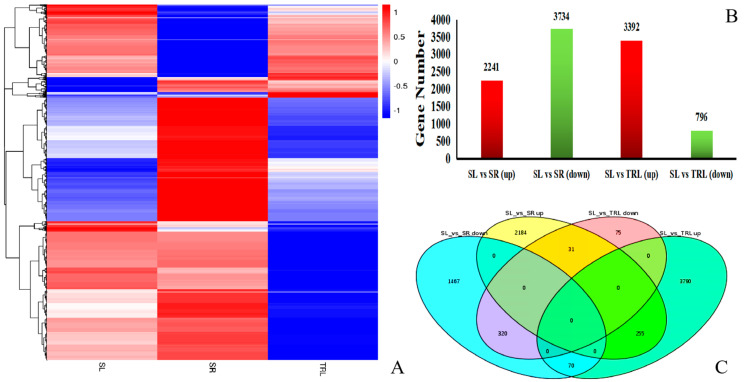
Differentially expressed gene (DEG) hierarchical clustering. (**A**) Heat map showing genes with differential expression (fold ≥ 1, false discovery rate (FDR) ≤ 0.001) among the three replicates of the SR (root), SL (sporophyll), and TRL (sporophyll with glandular trichomes removed). Expression values are log2-transformed and median-centered by gene. Scaled log2 expression values are shown, with red and blue indicating high and low expression, respectively. (**B**,**C**) Differences in gene expression in three tissues.

To identify the differences between each stage of the SR, SL, and TRL tissue samples, as well as differences according to tissue, a cluster map was drawn showing the correlations between biological replicates (Figure S4). A high correlation was indicated by a large correlation coefficient, as was the significance in the distinction among the different stages. Genes exhibiting significant differential expression (differentially expressed gene (DEG): −1 ≤ DEG ≤ 1f; false discovery rate (FDR) ≤ 0.001) were identified. Hierarchical clustering through priority DEGs revealed that there were 11,595 differentially expressed genes (Figure 1A). There were 2241 upregulated and 3734 downregulated genes in SL vs. SR, and 3392 upregulated and 796 downregulated genes in SL vs. TRL (Figure 1B). The Venn diagram of gene expression in the different tissues in Figure 1C shows that 255 genes in SL vs. SR and SL vs. TRL were upregulated, 320 in SL vs. SR and SL vs. TRL were downregulated, 21 were upregulated in SL vs. SR and downregulated in SL vs. TRL, and 70 were upregulated in SL vs. TRL and downregulated in SL vs. SR.

### 2.3. Differentially Expressed Gene Analysis of GO Enrichment

**Figure 2:**
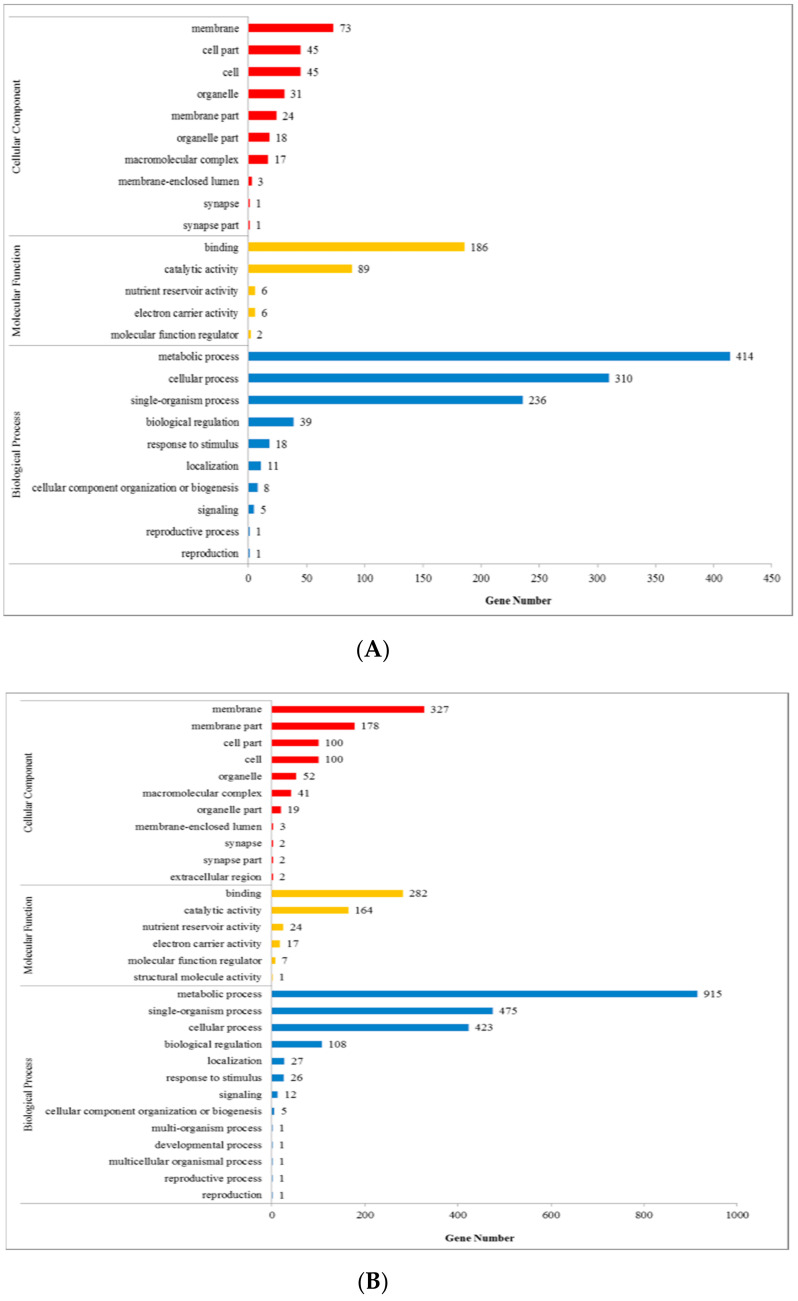
Gene ontology (GO) classification: (**A**) SL vs. SR, (**B**) SL vs. TRL. The *y*-axis represents the next-level GO term of the three major GO categories. The *x*-axis represents the number of genes annotated to the term (including the subterm) and proportion of the total number of annotated genes. The three different GO categories include biological processes, cell components, and molecular functions.

The GO database is a system that integrates the standard biological annotation of all species. We sought to explore whether tissue-specific gene clusters were enriched according to specific gene ontologies (GOs) relating to molecular function, cellular components, or biological processes for the SL, TRL, and SR gene ontology classes (terms with corrected *p* ≤ 0.01). In this database, the matching unigenes are divided into several layers, and the lower the level of the node, the more specific the represented function. In SL vs. SR, the “cell part” (45, 17.4%), “membrane” (73, 28.3%), and “cell” (45, 17.4%) had the highest numbers of matches in the cellular component. For molecular function, “catalytic activity” (89, 30.8%) and “binding” (186, 64.4%) were significantly higher than the other categories. For biological processes, “metabolic process” (414, 39.7%), “cellular process” (310, 29.7%), and “single-organism process” (236, 22.6%) were the most enriched. In SL vs. TRL, the “membrane” (327, 39.6%), “membrane part” (178, 21.6%), “cell part” (100, 12.1%), and “cell” (100, 12.1%) had the highest numbers of matches in the cellular component. In terms of molecular function, “catalytic activity” (164, 57%) and “binding” (282, 33.1%) were remarkably higher than other categories. As for biological processes, "metabolic processes" (915, 14.1%), “cellular process” (423, 21.2%), and “single-organism process” (475, 23.8%) exhibited the greatest enrichment (Figure 2).

### 2.4. Kyoto Encyclopedia of Genes and Genome (KEGG) classification

**Figure 3:**
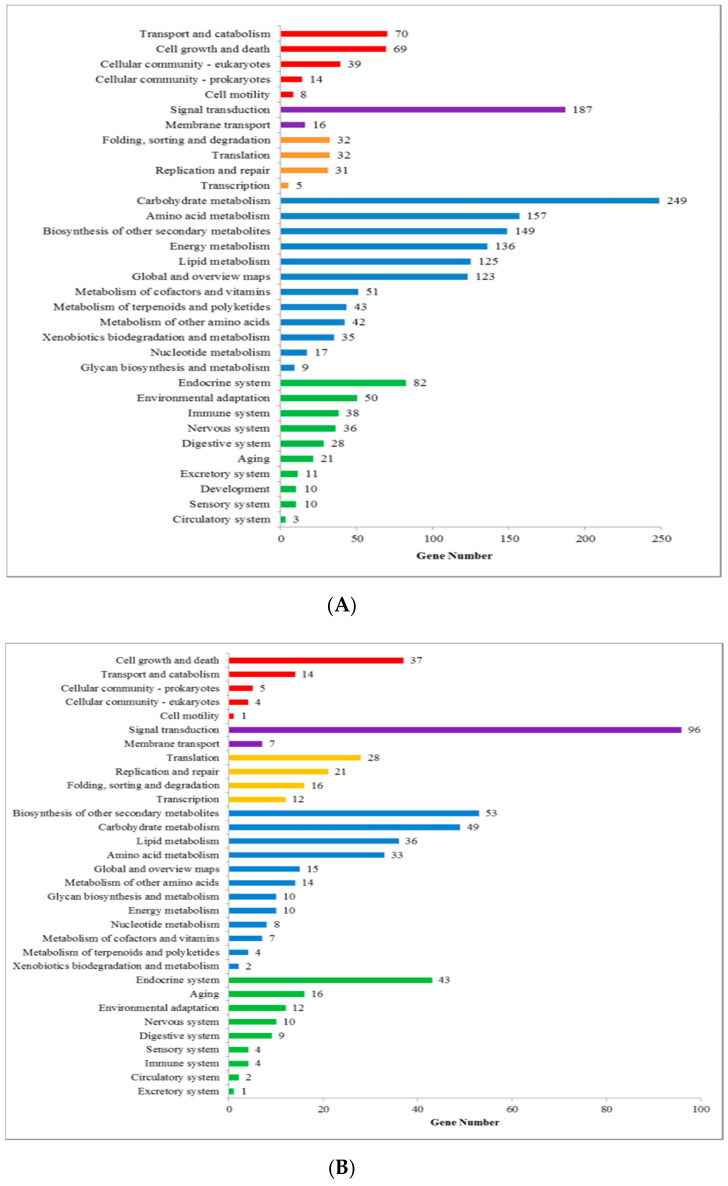
Kyoto Encyclopedia of Genes and Genomes (KEGG) classification: (**A**) SL vs. SR, (**B**) SL vs. TRL. The *y*-axis represents the names of the 26 groups of the KEGG. The *y*-axis represents the ratios of the number of annotated genes for each group to the total numbers of annotated genes. Different colors are used to distinguish different enrichments.

KEGG analysis showed that 583 genes were distributed across 33 pathways in SL vs. TRL (Figure 3). These genes could be divided into five groups according to the KEGG metabolic pathways: cellular process, environmental information processing, genetic information processing, metabolism, and tissue system. The major pathways included “signal transduction” (96, 16.47%), followed by “biosynthesis of other secondary metabolites” (53, 9.09%) and “carbohydrate metabolism” (49, 8.41%). In SL vs. SR, the KEGG analysis of the unigenes revealed 1928 unigenes. The major pathways were “carbohydrate metabolism”, (249, 12.91%) followed by “signal transduction” (187, 9.67%) and “amino acid metabolism” (157, 8.14%).

### 2.5. D. fragrans Transcription Factor Expression within Different Tissues

**Figure 4:**
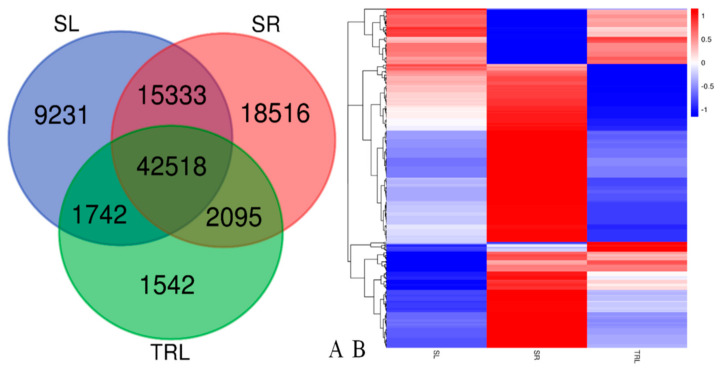
(**A**) Three-way Venn diagram showing the numbers of shared and unique expressed genes across the three tissues. (**B**) Differentially expressed transcription factors. Heat map showing transcription factors with differential expression (fold ≥ 1, FDR ≤ 0.001) among the three replicates of the SR (root), SL (sporophyll), and TRL (sporophyll with glandular trichomes removed). Expression values are log2-transformed and median-centered by gene. Scaled log2 expression values are shown, with red and blue indicating high and low expression, respectively.

SR appeared to have the highest occurrence of tissue-specific expression (18,516 genes), followed by SL (9231) and then TRL (1245) (Figure 4A). Apart from 42,518 genes that were shared among the three tissues, the highest number of shared genes was 15,333 between SR and SL, followed by 2095 between SR and TRL and then 1724 between SL and TRL. We next inspected the expression patterns of TFs genes among the three tissues. Using only significantly differentially expressed transcription factors, hierarchical clustering revealed distinctive expression profiles for each tissue (Figure 4B).

**Figure 5:**
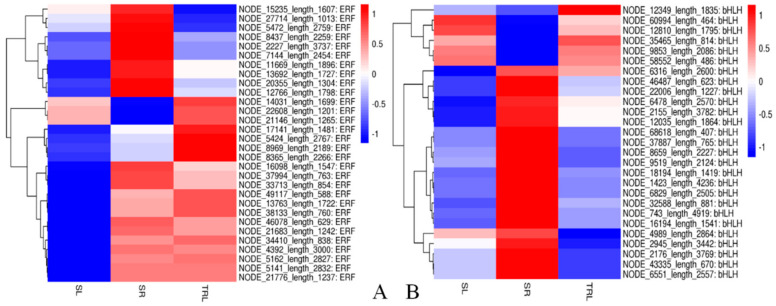
(**A**) ERF transcription factor thermogram; (**B**) bHLH transcription factor thermogram. Heat map showing that the two most significant transcription factors, ERF and bHLH, were expressed with differential expression (fold ≥ 1, FDR ≤ 0.001) among the three replicates of the SR (root), SL (sporophyll), and TRL (sporophyll with glandular trichomes removed). Expression values are log2-transformed and median-centered by gene. Scaled log2 expression values are shown, with red and blue indicating high and low expression, respectively. Indicated on the right are the gene IDs.

After identifying the transcription factors exhibiting significant differential expression (we searched for transcription factor clusters exhibiting tissue-specific expression. The results show that the ERF and bHLH transcription factors were the two types showing the most distinct expression patterns among the three examined tissues (Figure 5A,B). The bHLH transcription factors mainly include *BIM2* (NODE_1423_length_4236), which encodes *BES1-INTERACTING MYC-LIKE 2*, a *PAR1* (*PHYTOCHROME RAPIDLY REGULATED 1*)-interacting protein that positively modulates shade-avoidance syndrome. Specifically, *bHLH63* (NODE_22006_length_1227) can trigger flowering in response to blue light. *FAMA-like* (NODE_32588_length_881), together with *MYB88* and *MYB124*, ensures that stomata contain only two guard cells (GCs) by enforcing a single, symmetric precursor cell division before stomatal maturity. With SPCH and MUTE, FAMA-like (NODE_32588_length_881) regulates stoma formation. The ERF transcription factors mainly include *ethylene-responsive transcription factor 7-like* (NODE_13763_length_1722). Others found included the *ethylene-responsive transcription factor ABR1-like isoform X2* (NODE_5162_length_2827), an ABI1-mediated abscisic acid (ABA) response gene, and ethylene-responsive transcription factor *ERF113-like* (NODE_5424_length_2767), which regulates stomatal closure and antioxidant enzyme activity through the ABI1-mediated abscisic acid (ABA) signaling pathway. Other members act as transcriptional activators to regulate the components involved in stress signal transduction pathways and plant development.

**Figure 6:**
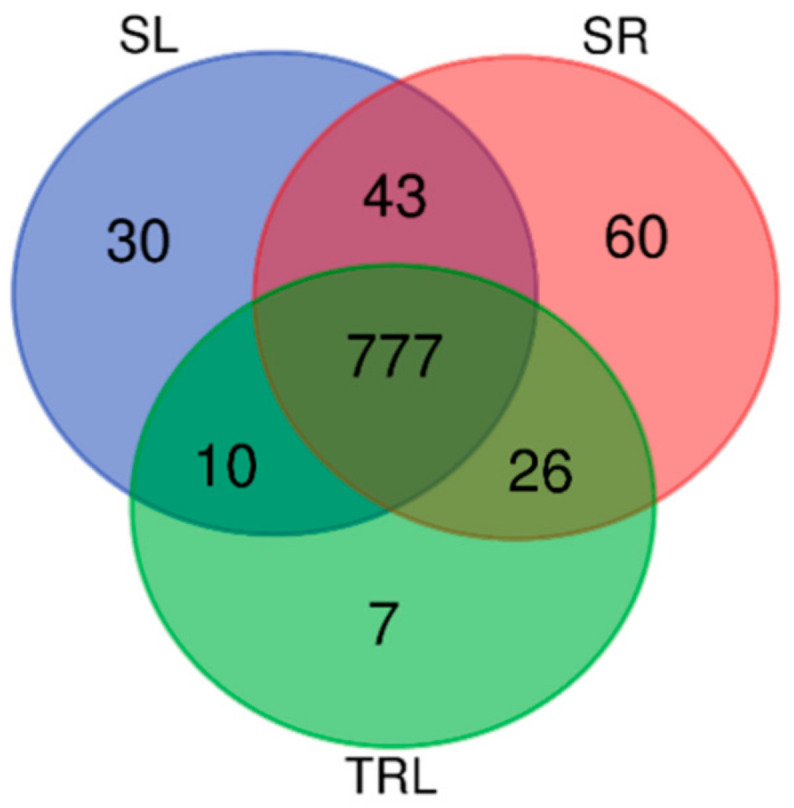
Three-way Venn diagram showing the numbers of shared and unique expressed transcription factors across the three tissues: SR (root), SL (sporophyll), and TRL (sporophyll with glandular trichomes removed).

Of the 953 TF genes annotated, 12.6% were expressed in at least one tissue and 81.5% in all three tissues (Figure 6). The SR appeared to have the highest occurrence of tissue-specific expression (60 transcription factors), followed by the SL (30) and TRL (7), including some well-studied examples. In the SL, 30 transcription factors, mainly related to plant development, secondary metabolism, hormone response, and inorganic salt transport, were specifically expressed. These transcription factor genes included *putative constans-like protein* (NODE_21731_length_1239), *MADS-domain transcription factor* (NODE_22852_length_1190), *LOB domain-containing protein 41-like* (NODE_25987_length_1067), *NAC domain-containing protein 86-like* (NODE_37861_length_765), *transcription factor DUO POLLEN 1* (NODE_64244_length_437), *AIG1* (NODE_64255_length_437), and *homeobox protein knotted-1-like 13 isoform X1* (NODE_7223_length_2439), which may participate in sporangial development; in addition, *AP2/ERF* (NODE_17285_length_1472) may function as a negative regulator of *D. fragrans* growth and development. *ARFF_ARATH auxin response factor* 6 (NODE_38417_length_754) and *ARF-L1 protein* (NODE_34396_length_838) are related to auxin response, and the *ethylene-responsive transcription factor RAP2-12-like* (NODE_37093_length_779) is related to ethylene. The *ERF78_ARATH ethylene-responsive transcription factor 4* (NODE_53904_length_532) is involved in the regulation of gene expression by stress factors and components of stress signal transduction pathways. It also includes *squamosa promoter-binding-like protein 7* (NODE_82760_length_331), acting coordinately with *HY5* to regulate *miR-408* and its target genes in response to changes in light and copper conditions. The *dehydration-responsive-element-binding protein 2D* (NODE_34268_length_841) can mediate high-salinity-induced transcription, *transcription factor Y subunit C-2-like* (NODE_30657_length_928) can adjust Ca^2+^ transport, and DNA*J-class molecular chaperone with C-terminal Zinc finger* (NODE_27104_length_1031) is related to metal binding. In the SR, 60 transcription factors are specifically expressed; 15 of these are hybrid proteins, while the others are related to plant morphological development, ubiquitination, and the cell cycle. For example, the *PLET2_ARATH AP2-like ethylene-responsive transcription factor PLT2* (NODE_74565_length_371) is essential for root quiescent center (QC) and columella specification, stem cell activity, and the establishment of the stem cell niche during embryogenesis. The *class III homeodomain-leucine zipper protein* (NODE_64330_length_437), *B3 domain-containing transcription factor NGA1-like* (NODE_79315_length_347), *B3 domain-containing protein At3g19184* (NODE_11730_length_1890), *BEL1-like homeodomain protein 1* (NODE_3776_length_3170), and *ethylene-responsive transcription factor 2-like* (NODE_68637_length_406) can regulate lateral root formation and the *meristem. Trihelix transcription factor ASIL2-like* (NODE_11049_length_1955) is related to embryonic development*; calmodulin-binding transcription activator 3-like isoform X*2 (NODE_22697_length_1196) may have similar functions; and baby boom (NODE_42351_length_686) promotes cell proliferation, differentiation, and morphogenesis, especially during embryogenesis. *E3 ubiquitin-protein ligase makorin-2* (NODE_14800_length_1638) and *putative transcription factor pbx* (NODE_23838_length_1151) are related to ubiquitination. The *class III HD-Zip protein HDZ2* (NODE_87491_length_312) is related to lipid binding. *Cell division cycle 5-like* (NODE_71830_length_386) is a component of the MAC complex that probably regulates defense responses through transcriptional control, and would thereby be essential for *D. fragrans*’ innate immunity. *Calmodulin-binding transcription activator 3-like isoform X2* (NODE_22697_length_1196) is possibly related to embryonic organ development. In the TRL, the *homeobox protein knotted-1-like 1* (NODE_53201_length_540) interacts with auxin and AS1, which results in the promotion of leaf fate, and *Dof2* (NODE_8258_length_2282) may transactivate seed storage protein genes in developing seeds; however, in *D. fragrans*, *Dof2* may instead be related to spore development. *WRKY transcription factor 12* (NODE_12261_length_1843) is involved in aging, as well as biological and abiotic stress responses by regulating various plant hormone signaling pathways. *Zinc finger protein 36* (NODE_17837_length_1440) can regulate ABA-induced hydrogen peroxide production and antioxidant defenses. *Zinc finger CCCH domain-containing protein 35* (NODE_20185_length_1313) and *GATA domain-containing protein* (NODE_46526_length_622) play roles in metal ion binding and zinc ion binding, respectively.

### 2.6. Transcription Factors Related to Trichome Development in D. fragrans

**Figure 7:**
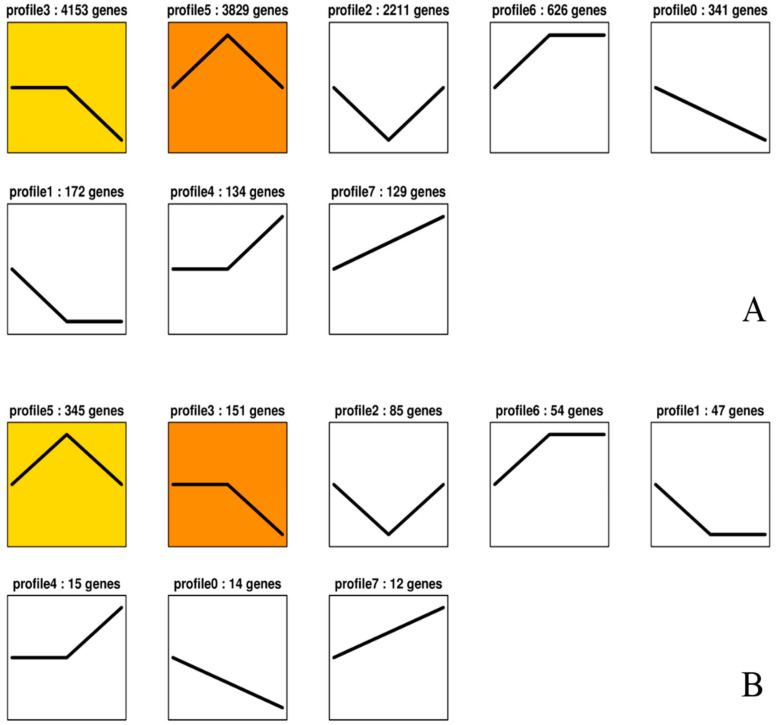
(**A**) Overall trend map of numbers of differentially expressed genes; profile analysis of the union of differential genes. (**B**) Overall trend map of gene numbers displayed by transcription factors; trend analysis of differentially expressed genes annotated as transcription factors. At the top of the graphs are the IDs of the profiles and the numbers of genes in the trends. Graphs with colors show significant enrichment trends, and different colors distinguish different trends. The graphs without color show nonsignificant enrichment trends.

Despite some TF genes being detected in all three tissues, the results imply that these TF genes and other genes still show a differential expression profile (Figure 7A,B), which may be related to their function in the regulation of glandular trichome growth and development. Since TF genes are thought to serve as key gene expression regulators, their patterns may indicate their involvement in various tissue-specific gene regulatory networks. Although the developmental regulation of fern trichomes has not been reported, *D. fragrans* does contain dense trichomes. Current studies have shown that the development of trichomes in different plants is regulated by transcription factors that are similar between different plants. We compared the sequence fragments in the transcriptome with those found to be related to trichogenesis in other species, as shown in Table 2.

Apart from the 777 transcription factors that were shared among the three tissues, the highest number of shared transcription factors was 43 between SR and SL, followed by 26 between SR and TRL, and 10 between SL and TRL. It is worth noting that the 26 transcription factors shared by SR and TRL are related to trichome development—for example, *MYC4* (NODE_12035_length_1864), which is involved in the regulation of the jasmonic acid (JA) gene. *Zinc finger protein 8* (NODE_43095_length_674) regulates the initiation of trichome growth in *Arabidopsis thaliana* [21]. *MYB80* (NODE_10729_length_1989) is related to the development of trichomes, and can negatively regulate the overlap and branching of trichomes. The R2R3–MYB transcription factor *MYB2* (NODE_9201_length_2161) regulates the initiation of cotton fiber growth in *Gossypium hirsutum* [22]. Some transcription factors confer resistance to biological stress, such as *EPF-type Cis2-His2 zinc finger transcription factor* (NODE_36794_length_786) and *WRKY transcription factor 46* (NODE_41105_length_707). The R2R3-MYB transcription factor *MYB9* (NODE_88329_length_309), related to trichome development, is expressed only in SR, so it may not be related to the development of trichomes in *D. fragrans*. Among the 10 transcription factors shared by SL and TRL, the transcription factor *bHLH49* (NODE_35465_length_814) is a transcriptional activator involved in cell elongation. The other genes, such as *BEL1-like homeodomain protein* 1 (NODE_48395_length_597) and *BEL1-like homeodomain protein 2* (NODE_64557_length_435), can establish leaf shape. *KNAT4_ARATH Homeobox protein knotted-1-like 4* is a homeodomain protein of the KNOX class I family, which has been shown to play a role in shoot apical meristem development. Genes related to flower development were also identified, such as *transcription factor PIF4* (NODE_2431_length_3644), light-regulated *zinc finger protein 1* (NODE_83844_length_327), and *NFYB6_ARATH Nuclear transcription factor Y subunit B-6* (NODE_23238_length_1174), which play vital roles in the regulation of seed maturation in *Arabidopsis* [23]. 

Most genes expressed only in leaves are related to flower and seed development. These include *BLH1_ARATH BEL1-like homeodomain protein 1* (NODE_3776_length_3170), *MAD50_ORYSJ MADS-box transcription factor 5* (NODE_26719_length_1043), and *ARFB_ARATH Auxin response factor 2* (NODE_7811_length_2347). In addition, *MYB98* (NODE_17251_length_1474) controls the development of specific features within the synergid cell during female gametophyte development, and is also expressed in trichomes and endosperm.

We further analyzed the differentially expressed genes and transcription factor gene expression trends. The sequence NODE_11206_length_1939 is the *MIXTA* of MYB transcription factors, which is related to trichome formation in *Anthurium japonicum* [24]. In this study, the expression pattern of *MIXTA* was relatively high in SL, moderate in roots, and very low in TRL, indicating that *MIXTA* might be related to the trichomes of *D. fragrans.* In addition, its homologous gene *MYB106* (NODE_11206_LENGTH_1939) is also associated with the occurrence of trichomes, and the similarity in expression patterns with those of *MIXTA* consistently indicates that they may have the same function. Moreover, *MYB106* is involved in the negative regulation of trichome branching. The *MIXTA*-like MYBs regulate the biosynthesis of cutin nanoridges and wax accumulation by promoting the expression of related genes [25]. Notably, some genes that encode the *homeodomain leucine zipper class I* (HD-Zip I) *HOX21* (NODE_7286_length_2427), *HOX5* (NODE_8227_length_2288), and *HOX4* (NODE_30917_length_922) transcriptional activators, which are involved in leaf development, have the same expression pattern, which is relatively high in the SL.

### 2.7. Gene Regulatory Network Construction

**Figure 8:**
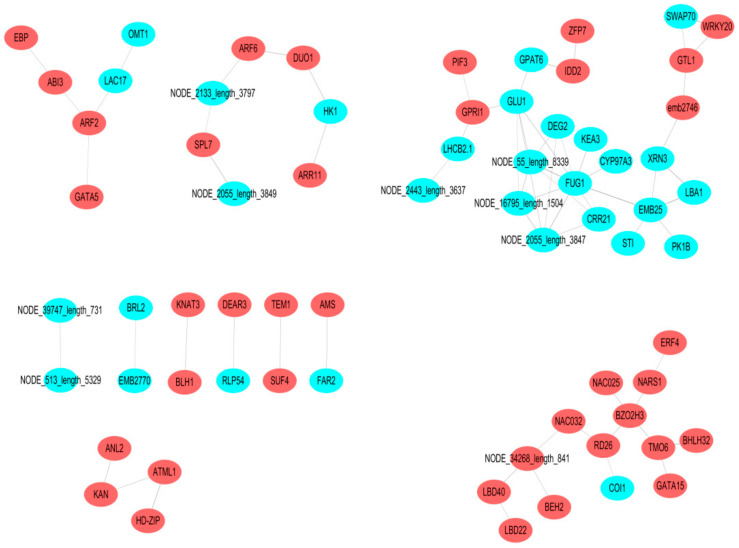
Prediction of protein interactions; red denotes differentially expressed transcription factor genes is highly expressed in the SL, while light blue denotes transcription factors is highly expressed in the SL,. Line thickness indicates the strength of support by the data. The thicker the line, the stronger the possibility of protein interaction.

We found several genes and transcription factor genes to be highly expressed in the SL and thus, conducted transcription factor genes differentially expressed analyses (Figures S5 and S6). The 36 major unigenes belong to MYB, followed by five unigenes belonging to bHLH, five to C2H2, four to HD-ZIP, and three to WRKY. The highly expressed prediction interacting proteins in leaves were found to mainly be comprised of the following genes. First, auxin-responsive genes, such as the auxin response factor 2 colony, include ABI3, which is pertinent to seed maturation (Figure 8) by regulating the transition between embryo maturation and early seedling development. Second, the putative seed-specific *transcriptional activator LAC17 likely* contributes to lignin biosynthesis, and hence, cell wall biosynthesis. Third, *GATA5* is involved in regulating carbon and nitrogen metabolism. Overall, these genes may regulate the functions of spore and leaf cell development. The fourth cluster includes proteins involved in photoresponse, chloroplasts, and cell development, as well as cell-silencing clusters associated with *GLU1*, *FUG1*, *EMB25*, and *GTL1* gene interactions. Gene clusters interacting with the transcription factor Col1 are related to sporocyst development.

*ARATH Homeobox-leucine zipper protein MERISTEM L1* (NODE_908_length_4702) was highly expressed in the SL, and encodes a homeobox protein similar to the homeobox–leucine zipper protein GLABRA 2 in *Arabidopsis thaliana*. Gene *HD-ZIP* (NODE_33044_length_869) of the *homeodomain GLABROUS 2* encodes a homeobox–leucine zipper family protein belonging to the HD-ZIP IV family, similar to *ATML1*, Mutants have trichomes that appear glass-like under a dissecting microscope compared to the wild-type trichomes, although interaction may occur in both types. The *homeobox–leucine zipper protein anthocyaninless 2* (NODE_25007_LENGTH_1104) encodes a homeodomain protein of the HD–GLABRA2 group, involved in the accumulation of anthocyanins, root development, and regulating tissue polarity in *A. thaliana*. *KAN* (NODE_56983_length_501) is required for abaxial identity in both leaves and carpels, and appears to be involved in the development of the carpel and outer integument of the ovule. Interaction may also occur between *ANTHOCYANINLESS* and *KAN*. 

### 2.8. Validation of DEGs by qPCR

Eight genes were selected for validation based on the results acquired from the KEGG pathway enrichment analysis. As shown in Figure S6, the trends of their gene expression levels and FPKM values tended to be the same.

## 3. Materials and Methods

*D. fragrans* spores were collected from lava rock near Bagua Lake, Wudalianchi, Heilongjiang, China (48.733334 N, 126.167966 E), within a 30 m radius during July 1–6, 2016, with permission from the government. To ensure the uniformity of samples, we used *D. fragrans* that was grown at 25 °C with a 16/8 h photoperiod over 2 years. We subsequently selected three tissues of this *D. fragrans* to investigate gene regulation and glandular trichome development. We selected the roots, sporophylls, and sporophylls with the glandular trichomes of *D. fragrans* removed; for their removal, the trichomes were wiped with a brush dipped in liquid nitrogen. A transcriptome database was established using Illumina sequencing to further elucidate the genes of the fern (Beijing Genomics Institution, Shenzhen, China).

To characterize the transcriptome, three biological replicates were obtained for each tissue (SR1, SR2, SR3; SL1, SL2, SL3; and TRL1, TRL2, TRL3). For real-time quantitative PCR (qPCR) analysis, the same samples originally used for RNA-seq were used with three biological replicates and three technical replicates for each. All experimental materials were transplanted from the tissue culture, and the optical microscope pictures were taken in the laboratory.

### 3.1. RNA Isolation and Qualification

Total RNA was isolated from the samples using a TIANGEN RNAprep Pure Plant Kit, according to the manufacturer’s instructions. A NanoDrop 2.0 Spectrophotometer and Agilent 2100 Bioanalyzer were used to characterize the RNA purity and concentration prior to transcriptomic sequencing. Transcriptomic data were obtained from nine RNA-seq libraries of three tissue samples with three biological replicates.

### 3.2. Library Preparation for Transcriptomic Sequencing

Sequencing libraries were generated using NEBNext^®^ Ultra™ RNA Library Prep Kit for Illumina^®^ (#E7530L, NEB, MA, USA). The mRNA was enriched using oligo(dT) magnetic beads. Fragmentation buffer was then added to randomly fragment the mRNA. Firstly, cDNA was prepared from 1 microgram total RNA with random hexamers, according to the manufacturer’s instructions. Then, the double-stranded cDNA was end-repaired and purified using AMPure XP beads, and tails and sequencing linkers were attached. AMPure XP beads were also used to select for the appropriate fragment sizes, and PCR enrichment was performed to obtain the cDNA libraries. A Qubit 2.0 Fluorometer and Agilent 2100 Bioanalyzer were used to determine the concentrations of the libraries and inserted fragment size. The effective library concentrations were determined by qPCR. Finally, the libraries were subjected to high-throughput Illumina sequencing (Illumina HiSeq^TM^ X TEN, San Diego, CA, USA).

### 3.3. Transcriptomic Analysis

#### 3.3.1. Read Mapping, Differential Gene Expression, and Clustering Analyses

Illumina reads were checked for quality using FastQC software. Trinity [26] was employed to assemble the sequences. The BLAST software was applied to compare the Unigene sequence with the Nr, Swiss-Prot, gene ontology (GO), Clusters of Orthologous Groups (COG)/euKaryotic Orthologous Groups (KOG), and eggNOG4.5 databases, while the Kyoto Encyclopedia of Genes and Genomes (KEGG) used Bowtie2 [27]. The software compared the reads of each sample with a unigene library, and evaluated the expression level by RESM software [28]. The abundance of unigene is expressed by fragment per kilobase per million mapped reads (FPKM). HTSeq v0.6.1 was used to count the read numbers mapped to each gene. To identify differential expression between samples and tissues, we applied both fold-change and false discovery rate (FDR) thresholds. Specifically, we set FDR to ≤0.001 and log2 (fold change) ≥ 2 to declare significance in line with previous recommendations. It is important to note a log2 (fold change) > 2 equates with a minimum four-fold change. 

#### 3.3.2. Gene Ontology (GO) Enrichment Analysis

Gene ontology (GO) enrichment was performed using the Singular Enrichment Analysis (SEA) tool of agriGO with the default settings. The significance of the differences in annotation frequencies was tested for each gene ontology term, at level 4, for biological process, molecular function, and cellular component, using Fisher’s exact test followed by a *p*-value adjustment for multiple testing, based on the Holm–Bonferroni method.

#### 3.3.3. Kyoto Encyclopedia of Genes and Genomes (KEGG) Pathway Database Enrichment Analysis

Metabolic pathway analysis was performed using the Kyoto Encyclopedia of Genes and Genomes Pathway database (KEGG: http://www.genome.jp/kegg/). We used the KOBAS software to test the statistical enrichment of differentially expressed genes (DEGs) in the KEGG pathways (*p*-value < 0.01). Pathway was significantly enriched in the differentially expressed Unigene.

#### 3.3.4. Gene Regulatory Network Construction Module Detection 

Pairwise Pearson correlations of gene expression across all the samples were calculated to generate a similarity matrix, which served as input for generating stress-specific co-expression networks, using R/WGCNA version 1.34. 

### 3.4. Trend Analysis of all Differentially Expressed Genes and Transcription Factors

The average FPKM values of the all differentially expressed genes and transcription factors in the three tissues were used as the starting data for expression pattern analysis. Specifically- or highly-expressed genes and transcription factors were only identified in leaves. The log2 standardization of the FPKM values (*p* < 0.05) was performed. Trend analysis was used to cluster the gene expression patterns of three groups of tissue samples: SR (root), SL (sporophyll), and TRL (sporophyll with glandular trichomes removed). The gene sets were then selected from the clustering results according to certain biological characteristics (e.g., the differentially expressed genes DEGs specific to SR). We used the STEM Short Time-series Expression Miner (http://www.cs.cmu.edu/~jernst/stem) and set the maximum number of model profiles to eight to analyze trends.

### 3.5. Real-Time qPCR Analysis

Ten transcription factor unigenes were chosen for validation by qPCR. The reference gene selected for normalization in this experiment was 18S rRNA [29]. The primers were designed with Primer Premier 5.0 (Table S2). The qPCR was performed with a Bio-Rad CFX-96 Real-Time PCR System (Bio-Rad, Hercules, CA, USA) with a final volume of 20 μL, containing 2 μL cDNA, 10 μL 26SYBR Premix Ex Taq (Takara Bio, Shiga, Japan), 0.4 μL each of 10 µM forward and reverse primers, and 7.2 μL RNase-free water. Thermal cycling was performed at 95 °C for 5 min, followed by 45 cycles of 95 °C for 5 s for denaturation and 56 °C for 25 s for annealing and extension.

This work provides the first comprehensive transcriptomic analysis of *D. fragrans* in the root and sporophyll. A total of 90,977 unigenes were identified, including 25,875 transcription factors, among which 50.65% were aligned to sequences in the Nr database. Although no *D. fragrans* reference genome sequence is available, the unigenes were annotated in the database. Eight unigenes were chosen for validation by qPCR, and the trends were largely in accordance with the transcriptomic data. A total of 1794 differentially expressed unigenes were found in the SL, SR, and TRL, including key trichome development genes, such as *DfMIXTA* and *DfAML1*. In summary, our results provide important insight into the complex transcriptional regulation and potential mechanisms underlying glandular trichome development, stress resistance, and secondary metabolism in *D. fragrans*. Some basic studies on the glandular trichomes of ferns were also discussed.

## 4. Discussion

In this study, global gene transcription in three different tissues of *D. fragrans* was studied using high-throughput sequencing. Through transcript profiling and comparative transcriptomic analysis, the gene expression patterns in diverse tissues were determined. The data included 90,977 unigenes, of which 25,875 were transcription factors. These may include be transcripts unique to each of the three examined tissues of *D. fragrans*. The differentially expressed genes shown in the heat mapping allow us to understand what is occurring in individual tissues. It is worth noting that some of the poorly repeated data should be studied further, rather than simply discounted.

Identifying genes expressed in different tissues provides baseline information for a broader understanding of tissue function and physiology. The study classified the collection of genes expressed in these three tissues and recorded crucial changes according to tissue. Moreover, we found that for the most abundant transcripts and transcription factor genes that exhibited tissue-specific expression, the results are consistent with expectations regarding their possible specific roles in different tissues. In particular, tissue specificity should be considered in the context of the tested tissue, and additional measurements in diverse tissues could not necessarily be used to confirm the observations. However, the GO enrichment profile captured from the transcriptome is in line with our expectations, which implies that this high-quality dataset is suitable for further analysis.

The transcription factors were differentially expressed in the different tissues. The numbers of transcription factors specifically expressed in the roots and sporophylls were 60 and 30, respectively, but only 7 in the sporophylls with glandular trichomes removed. This suggests that the regulation of development and metabolism by transcription factors is more prominent in the roots, while more transcription factors may be specifically expressed in glandular trichomes. Transcription factors related to sporangium development—such as putative constans-like protein, *MADS domain transcription factor*, *lob domain containing protein 41 like*, *NAC domain containing protein 86 like*, *transcription factor duo pollen 1*, *AIG1*, *homeobox protein known-1-like 13 isoform x1*, and *AP2/ERF*—were also found in the sporophyll, and may be expressed in the sporangium. The other transcription factors were mainly related to plant development, secondary metabolism, hormone response, and inorganic salt transport. In the SR, except for hybrid proteins, the other TF genes were related to plant morphological development, ubiquitination, and the cell cycle. In the TRL, the transcription factor genes were mainly associated with the promotion of leaf fate and spore development, and involved in aging, biological-, and abiotic-stress responses, hydrogen peroxide production, antioxidant defenses, and ion binding. The transcription factors specifically expressed in the glandular trichomes of *D. fragrans* may also be related to plant development, secondary metabolism, hormone response, and inorganic-salt transport.

We hypothesized that the expression of certain genes and transcription factor genes were affected by external stimuli, which may further influence multiple downstream genes and some unknown metabolic pathways [30]. We expect to pay more attention to these genes in the future. It has been documented that the function of uncharacterized genes can be inferred from their tissue-specific co-expression with known functional genes [19,31]. For instance, while the gene involved in pteridophyte trichome development has not yet been reported, it is known that *MIXTA* determines the occurrence of trichomes in *Antirrhinum majus*. In this study, *DfMIXTA* expression in the SL was significantly higher than that in the TRL. The trend of *DfMIXTA* expression was high in the SL and SR but low in the TRL, suggesting that this gene might be related to the development of trichomes in *D. fragrans*. *MYB106*, a gene homologous to *MIXTA*, plays the same role in angiosperms, such as *Arabidopsis thaliana*, and coexists in *D. fragrans*; thus, it may perform the same function in both plants. *Zinc finger protein 8* is one of the key transcription factors regulating trichome formation in angiosperms, while *zinc finger protein 1-like* regulates the initiation of trichome growth in *A. thaliana* [32,33]. Both transcription factors were highly expressed in the SR but not the SL in *D. fragrans*, suggesting that the regulation of glandular trichomes in ferns may be different from that in angiosperms. This further indicates that these transcription factors regulate root hairs or possibly the development of trichomes in *D. fragrans*. Although these genes are not expressed in sporophylls, they may play an important role in the early development of trichomes. Elucidating the details of the interaction of these genes with each other and how they function requires further research.

In addition, some genes are related to auxin, spore maturation, and photosynthesis. We predict that the *ARATH homeobox-leucine zipper protein MERISTEM L1* possibly interacts with the *HD-ZIP.* Similar to those in *Arabidopsis*, these complexes may initiate the expression of *DfAML1*, a gene encoding transcription factors, and the transformation of cells into trichomes, which is worthy of future study.

## 5. Conclusions

This work provides the first comprehensive transcriptomic analysis of *D. fragrans* in Root and Sporophyll. A total of 90,977 UniGenes were identified, including 25,875 transcription factors, among which 50.65% were aligned to sequences in the Nr database. Although no *D. fragrans* reference genome sequence is available, the UniGenes were annotated in the database. Eight UniGenes were chosen for validation by qPCR, and the trends were largely in accordance with the transcriptomic data. A total of 1794 differentially expressed UniGenes were found in SL, SR, and TRL, including key trichome development genes, such as *DfMIXTA* and *DfAML1*. In summary, our results provide important insight into the complex transcriptional regulation and potential mechanisms underlying glandular trichome development, stress resistance, and secondary metabolism in *D. fragrans*. Some basic studies on the glandular trichomes of ferns were also discussed.

## Figures and Tables

**Table 1:** The species and the tissues.

Species	Tissues	Abbreviation
*D. ragrans*	Root	SR
	Sporophyll	SL
	Sporophyll removed from glandular trichome	TRL

**Table 2:** Homologous sequences related to trichomes in *D. fragrans.*

Gene Family	Gene	Gene ID	Species	Homologous Sequence
MYB	*MYB0(GL1)*	AT3G27920	*Arabidopsis thaliana*	NODE_10793_length_1982
	*MYB1*			
	*MYB23*	AT5G40330	*Arabidopsis thaliana*	NODE_5558_length_2744
	*MYB2*	*AY626160*	*Gossypium arboreum*	NODE_9201_length_2161
	*MYB25*	*AF336283*	*Gossypium hirsutum*	NODE_12623_length_1810
	*MYB1*	*L04497*	*Gossypium hirsutum*	NODE_13457_length_1748
	*MYB7*	*AY518319*	*Gossypium hirsutum*	NODE_9733_length_2100
	*MYB106*	*AT3G01140*	*Arabidopsis thaliana*	NODE_11206_LENGTH_1939
	*MYB109*	*AJ549758*	*Gossypium hirsutum*	NODE_3492_length_3254
MYB	*MYB80*	*AT5G56110.1*	*Arabidopsis thaliana*	NODE_10729_length_1989
	*MYB9*	*AT5G67300*	*Arabidopsis thaliana*	NODE_88329_length_309
	*AaMIXTA*	KP195023.1	*Artemisia annua*	NODE_10723_length_1990
	*MIXTA*	AT5G15310.2	*Arabidopsis thaliana*	NODE_10792_LENGTH_1982
	*MIXTA*	X79108.1	*Antirrhinum majus*	NODE_11206_length_1939
	*MYBML8*	105961649/LOC105961649	*Erythranthe guttata*	NODE_648_length_5073
	*MYB16*	AT5G15310	*Arabidopsis thaliana*	NODE_64472_length_436
WD40	*TTG1*	*AT5G24520*	*Arabidopsis thaliana*	NODE_7598_length_2380
	*R*	*100126972/NC_024468.2*	*Zea mays*	NODE_2464_length_3629
WRKY	*TTG2*	*AT2G37260*	*Arabidopsis thaliana*	NODE_2852_length_3472
	*WRKY 46*	AT2G30590	*Arabidopsis thaliana*	NODE_41105_length_707
bHLH	*GL3*	*AT5G41315*	*Arabidopsis thaliana*	NODE_43335_length_670
	*EGL3*	*AT1G63650*	*Arabidopsis thaliana*	NODE_743_length_4919
	*MYC4*	AT4G17880	*Arabidopsis thaliana*	NODE_12035_length_1864
	*bHLH46*	AFU81789.1	*Papaver somniferum*	NODE_41105_length_707
	*bHLH49*	AT1G68920.3	*Arabidopsis thaliana*	NODE_35465_length_814
HD-Zip	*GL2*	AT1G79840	*Arabidopsis thaliana*	NODE_20550_length_1294
	*HOX21*	NC_029258.1	*Oryza sativa* subsp.	*NODE_7286_length_2427*
	*HOX5*	NC_029263.1	*Oryza sativa* subsp.	*NODE_8227_length_2288*
	*HOX4*	NC_029264.1	*Oryza sativa* subsp.	*NODE_30917_length_922*
	*ATML1*	AT4G21750	*Arabidopsis thaliana*	NODE_908_length_4702
	*PDF2*	AT4G04890	*Arabidopsis thaliana*	NODE_1662_length_4067
	*GLOXXY1*	LOC542154	*Zea mays*	NODE_11774_length_1887
	*ANTHOCYANINLESS 2*	AT4G00730.1	*Arabidopsis thaliana*	NODE_25007_LENGTH_1104
C2H2	*GIS*	*AT3G58070*	*Arabidopsis thaliana*	NODE_53584_length_536
ZFP5	*ZFP5*	*AT1G10480*	*Arabidopsis thaliana*	NODE_11304_length_1931
	*ZFP8*	AT2G41940	*Arabidopsis thaliana*	NODE_43095_length_674
	*ZFP6*	*AT1G67030*	*Arabidopsis thaliana*	NODE_53610_length_536
Importin-β	*SAD2*	*AT2G31660*	*Arabidopsis thaliana*	NODE_3732_length_3180

## Supplementary Materials

Supplementary materials can be found at https://www.mdpi.com/1422-0067/21/19/7296/s1.

## Abbreviations

The following databases are used in this manuscript:

COG	Clusters of Orthologous Groups
FPKM	fragment per kilobase per million mapped reads
GO	gene ontology
KEGG	Kyoto Encyclopedia of Genes and Genomes
KOG	Eukaryotic Orthologous Groups
MCMs	minichromosome maintenance proteins
NCBI	National Center for Biotechnology Information
