# Supplementary material for: Transcriptomic Analysis and Specific Expression of Transcription Factor Genes in the Root and Sporophyll of Dryopteris fragrans (L.) Schott
Source: Int J Mol Sci. 2020 Oct 2;21(19):7296. doi: 10.3390/ijms21197296 (PMC7583955; doi:10.3390/ijms21197296)
Supplement: Supplementary file 1 [file ijms-21-07296-s001.zip › Figure S.docx]

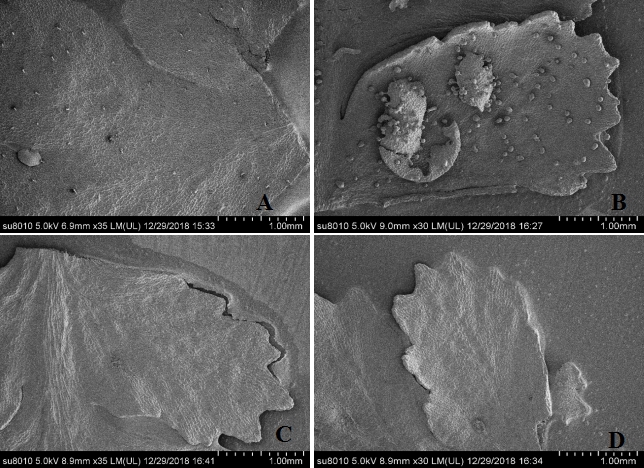


Figure S1. Sporophyll of *Dryopteris fragrans*

A: adaxial sporophyll; B: Sporophyll back;

C: Removing glandular trichome adaxial sporophyll;

D: Removing glandular trichome sporophyll back.

Figure S2. unigenes Length distribution

Transverse axis：unigenes length; Vertical axis：unigenes number.


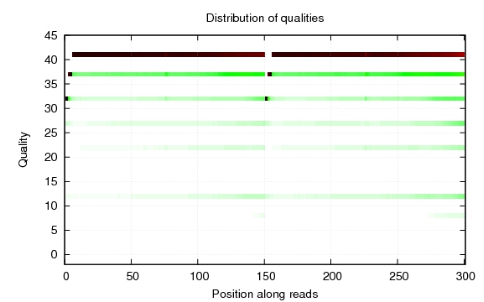


Figure S3. The box diagram of the quality distribution of each base sequence

The abscissa is the base position of reads (5'-> 3'), and the ordinate is the base mass value (Quality Score) of all reads at this site.The first 150 bp was the first end of the double-ended sequence, and the last 150 bp was the other end.


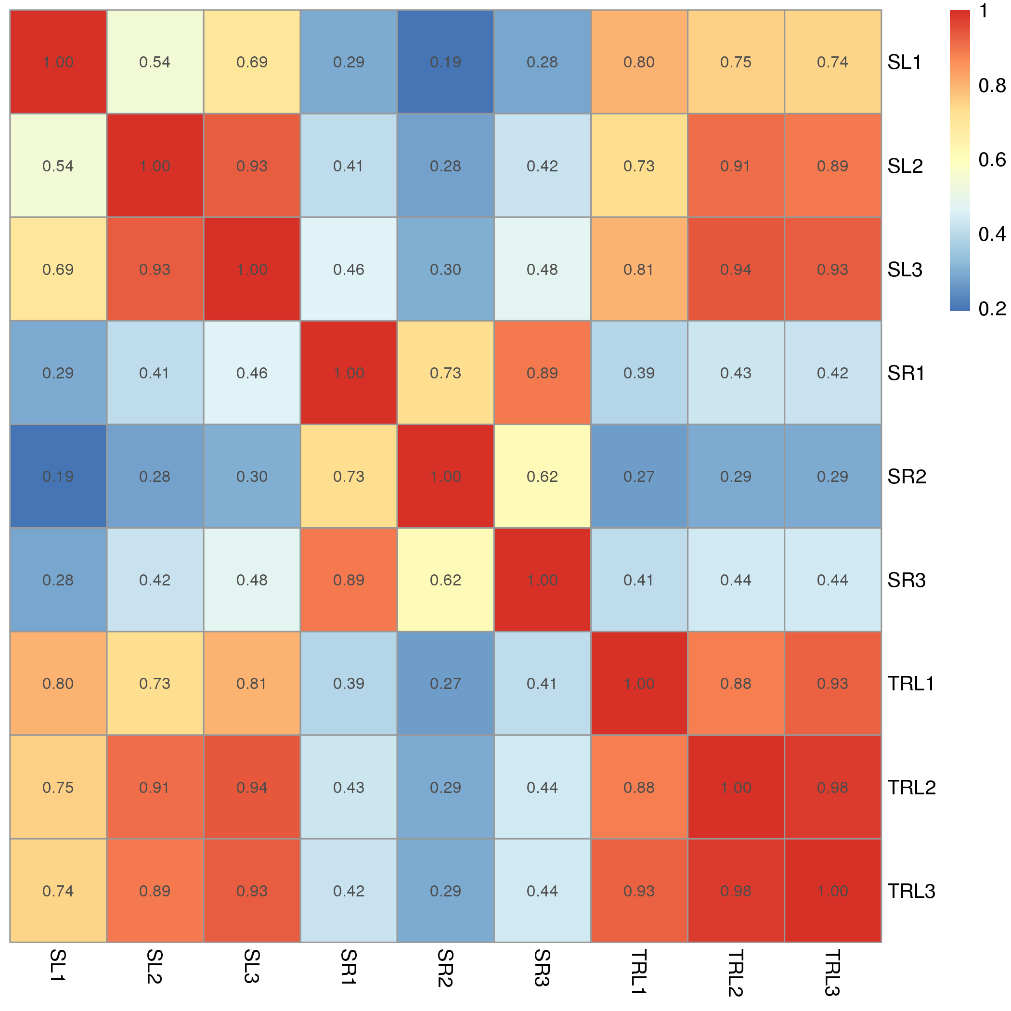


Figure S4. RNA-seq correlation detection

Level of agreement among the biological replicates of the three tissues. The heat map shows the hierarchically clustered Spearman correlations resulting from comparing normalized expression for all samples against one another. Sample clustering indicates the consistency between the biological replicates of each of the four tissues. SR (root), SL (sporophyll), and TRL (sporophyll removed from glandular trichome)


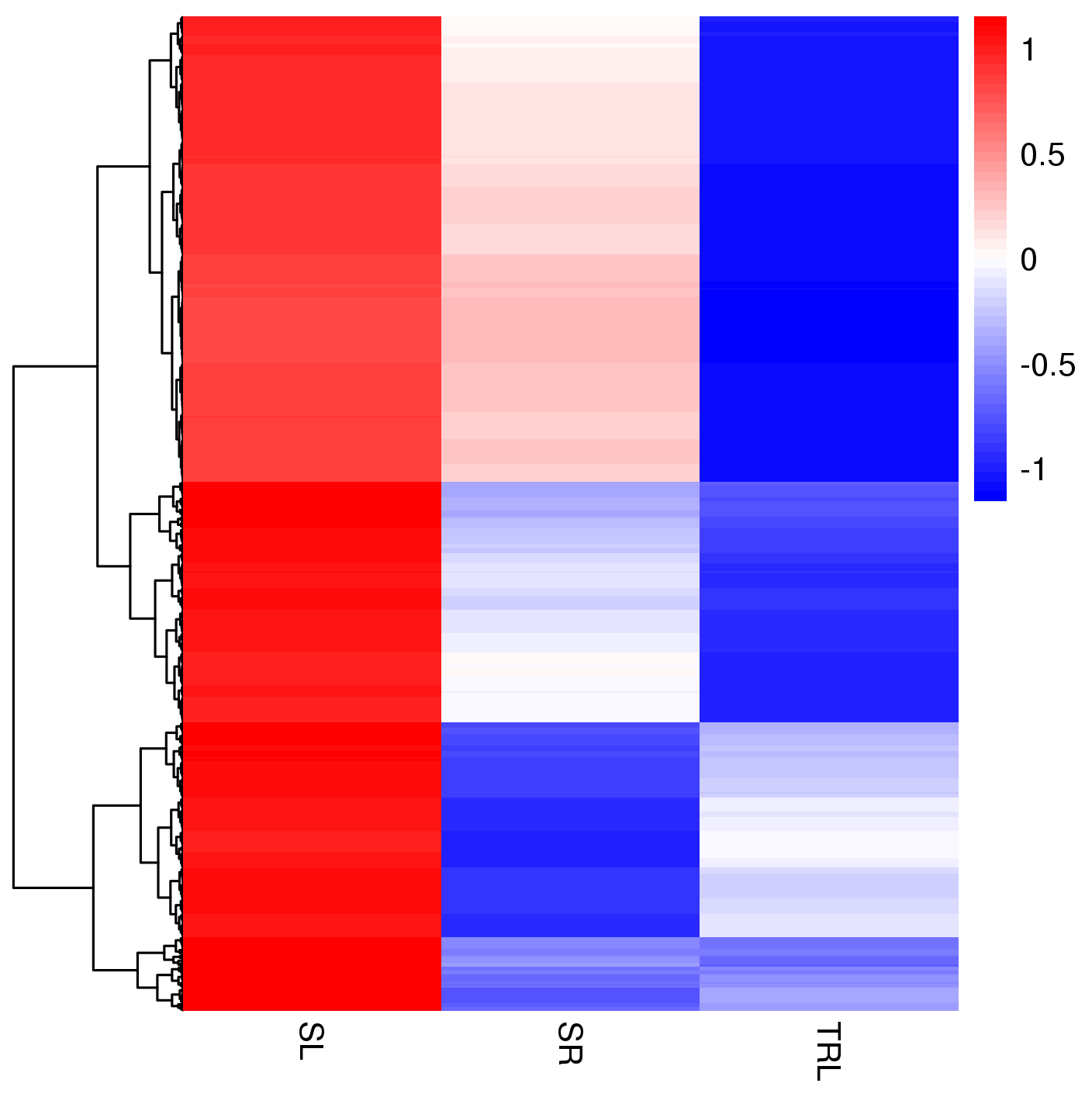


Figure S5. Differentially expressed gene attributed to SL(profile 0 and profile 1)


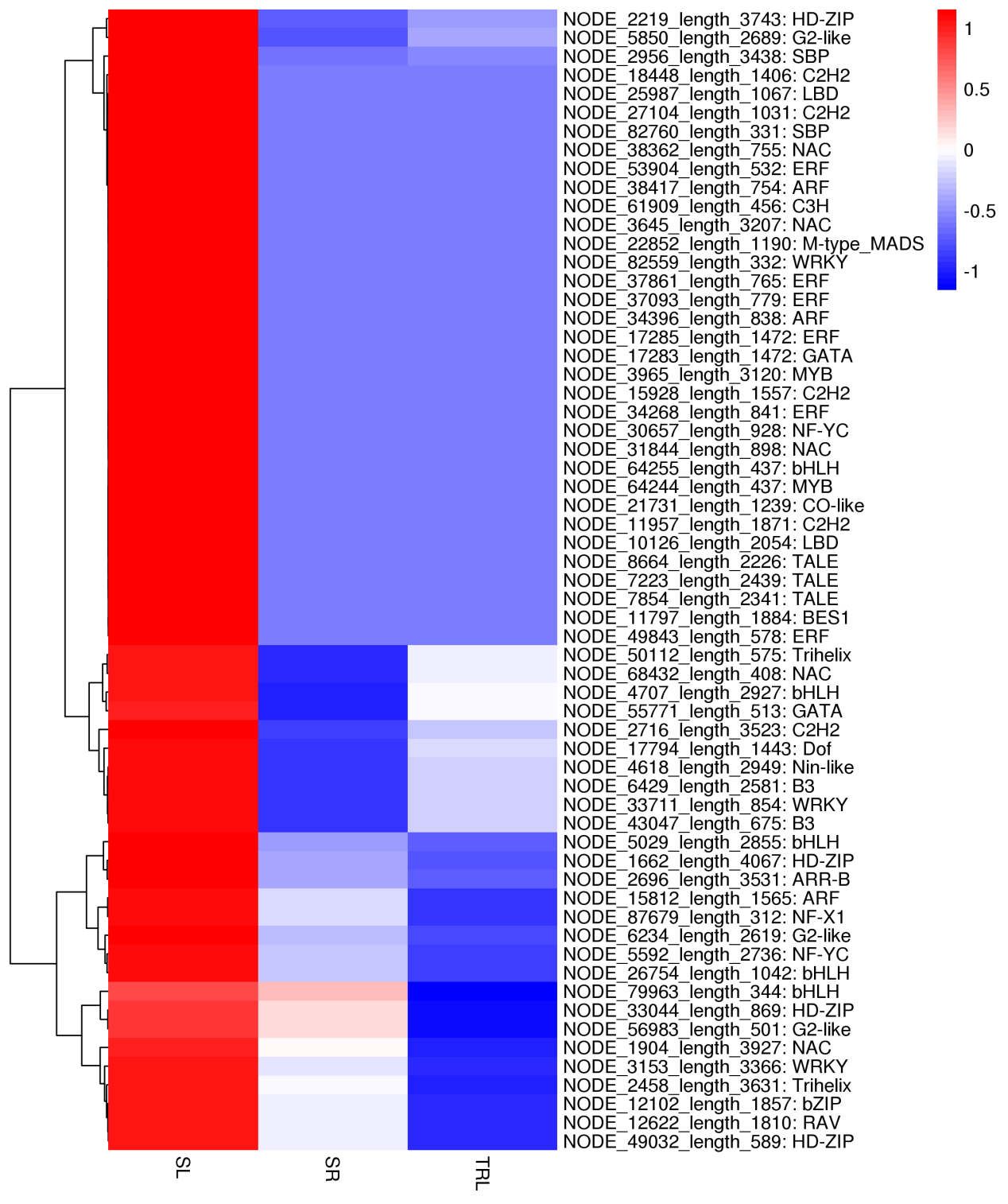


Figure S6. Differentially expressed Transcription factors attributed to SL(profile 0 and profile 1)


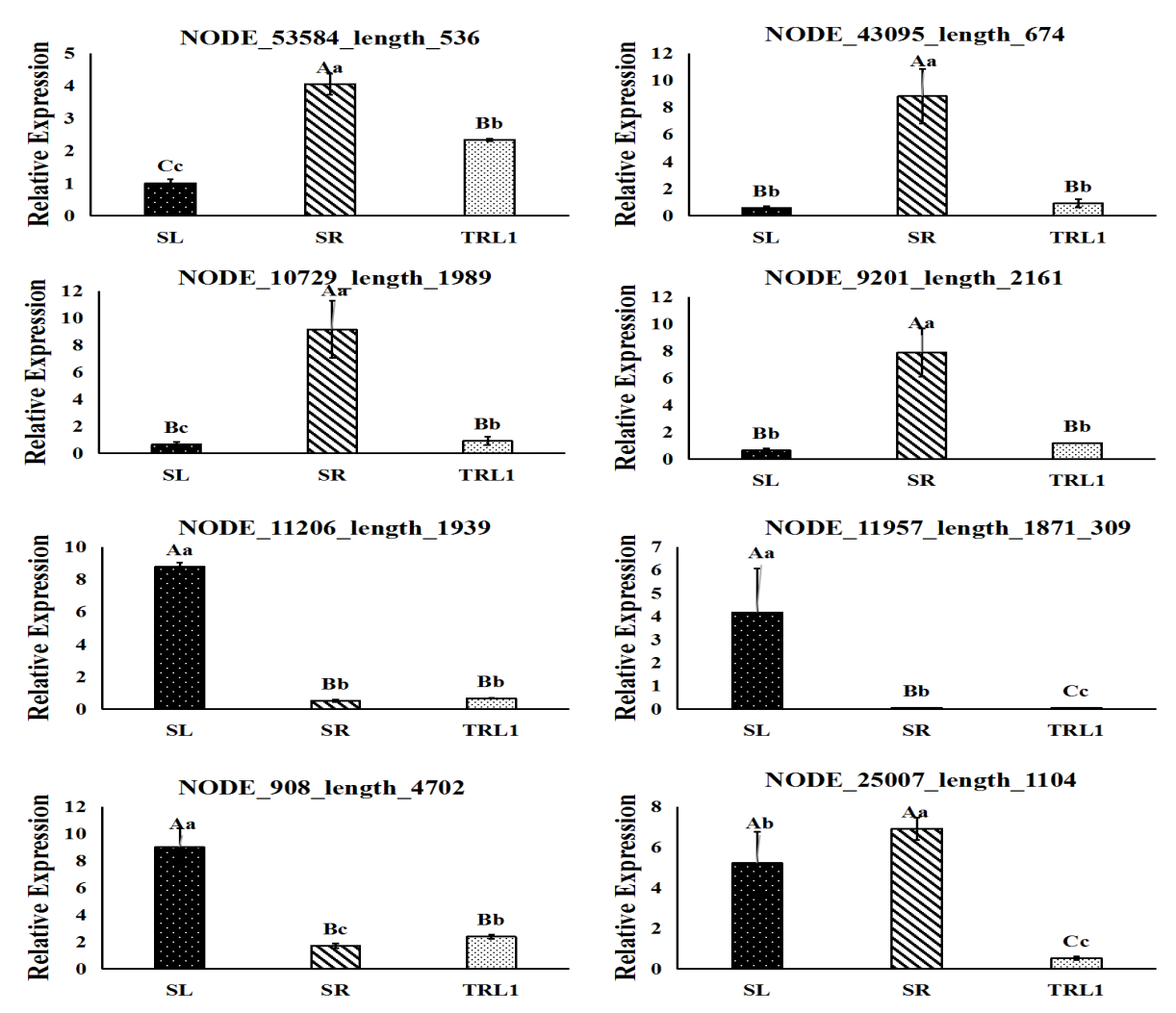


Figure S7. Real-Time qRT-PCR Analysis

SR (root), SL (sporophyll), and TRL (sporophyll removed from glandular trichome)
