# Supplementary material for: Transcriptomic Analysis and Specific Expression of Transcription Factor Genes in the Root and Sporophyll of Dryopteris fragrans (L.) Schott
Source: Int J Mol Sci. 2020 Oct 2;21(19):7296. doi: 10.3390/ijms21197296 (PMC7583955; doi:10.3390/ijms21197296)
Supplement: Supplementary file 1 [file ijms-21-07296-s001.zip › Table S1.docx]

Table S1. Illumina sequencing statistics. Data yield, number of obtained raw paired-end reads, number of mapped fragments for each RNA-Seq library.
